# Complementary religious and spiritual interventions in physical health and quality of life: A systematic review of randomized controlled clinical trials

**DOI:** 10.1371/journal.pone.0186539

**Published:** 2017-10-19

**Authors:** Juliane Piasseschi de Bernardin Gonçalves, Giancarlo Lucchetti, Paulo Rossi Menezes, Homero Vallada

**Affiliations:** 1 Instituto de Psiquiatria (LIM-23/ProSER), Faculdade de Medicina FMUSP, Universidade de Sao Paulo, Sao Paulo, SP, Brazil; 2 School of Medicine, Federal University of Juiz de Fora, Juiz de Fora, Brazil; 3 Departamento de Medicina Preventiva, Faculdade de Medicina FMUSP, Universidade de Sao Paulo, Sao Paulo, SP, Brazil; Universita degli Studi di Firenze, ITALY

## Abstract

**Objective:**

To examine whether religious and spiritual interventions (RSIs) can promote physical health and quality of life in individuals.

**Methods:**

The following databases were used to conduct a systematic review: PubMed, Scopus, Web of Science, EMBASE, PsycINFO, Cochrane, and Scielo. Randomized controlled trials that evaluated RSIs regarding physical health outcomes and/or quality of life in English, Spanish or Portuguese were included. RSI protocols performed at a distance (i.e. intercessory prayer) or for psychiatric disorders were excluded. This study consisted of two phases: (a) reading titles and abstracts, and (b) assessing the full articles and their methodological quality using the Cochrane Back Review Group scale.

**Results:**

In total, 7,070 articles were identified in the search, but 6884 were excluded in *phase* 1 because they were off topic or repeated in databases. Among the 186 articles included in *phase* 2, 140 were excluded because they did not fit the inclusion criteria and 16 did not have adequate randomization process. Thus, a final selection of 30 articles remained. The participants of the selected studies were classified in three groups: chronic patients (e.g., cancer, obesity, pain), healthy individuals and healthcare professionals. The outcomes assessed included quality of life, physical activity, pain, cardiac outcomes, promotion of health behaviors, clinical practice of healthcare professionals and satisfaction with protocols. The divergence concerning scales and protocols proposed did not allow a meta-analysis. RSIs as a psychotherapy approach were performed in 40% of the studies, and the control group was more likely to use an educational intervention (56.7%). The results revealed small effect sizes favoring RSIs in quality of life and pain outcomes and very small effects sizes in physical activity, promotion of health behaviors and clinical practice of health professionals compared with other complementary strategies. Other outcomes, such as cardiac measures and satisfaction with the protocols, revealed no evidence for RSIs. Regarding the quality of the selected articles according to the Cochrane Back Review Group Scale, the average score was 6.83 (SD = 9.08) on a scale of 11, demonstrating robustness in the studies.

**Conclusion:**

Clinical trials on RSIs demonstrated that they had small benefits compared with other complementary health therapies by reducing pain and weight, improving quality of life and promoting health behaviors. The lack of clinical trials that included biological outcomes and the diversity of approaches indicate a need for more studies to understand the possible mechanisms of action of RSIs and their roles in health care.

## Introduction

The current literature contains evidence about the impact of the religious/spiritual beliefs and practices on human health [1, 2]. Chronic health problems, such as hypertension, coronary artery disease and acquired immunodeficiency syndrome (AIDS/HIV+), seem to exhibit more control and stability in individuals with high levels of R/S practices [3, 4]. Data on different types of cancer also revealed better outcomes when patients have greater faith and spirituality during treatment [5].

Regarding mortality, a study demonstrated an increase in survival rates from 18% to 25% in people with higher levels of religiosity/spirituality (R/S) compared with other primary prevention in health prevention [6]. Even some health professionals reported improvements on their patient´s health, and were able to fulfill their patients’ spiritual needs in clinical practice when they were trained to increased awareness of the spiritual dimension in care [7]. These clinical findings highlight the need to integrate R/S into health care, aiming at complete physical, psychological, social and spiritual well-being [8], particularly in chronic patients.

Chronic health problems are a major public health issue due to the ageing of the world’s population, which is increasing the number of functionally compromised individuals [9]. In this context, patients tend to seek complementary therapies that may alleviate their discomfort and offer new possibilities of treatment. Complementary therapies are practiced by at least 40% of patients in the United States [10]. In fact, the prevention of these problems and the use of complementary medicine can reduce costs per individual [11] and improve quality of life [12]. Complementary therapies include religious/spiritual aspects, based on evidence of a positive association between R/S and health outcomes in general [1].

In recent years, the number of publications concerning R/S in the medical literature has been steadily increasing. In 1999, were published a total of 1,507 articles that included the terms *spiritual or *religio in at least one field of a bibliometric search. In 2013, the number of publications that included the same words had increased to 2,763 [13]. However, most studies are theoretical and observational, and some health professionals have doubts about how to approach R/S topics in clinical practice [14].

Thus, some authors have developed strategies to stimulate and develop the spiritual dimension of the patient to promote the clinical improvements identified in observational studies. These strategies are conventionally called religious and/or spiritual interventions (RSIs). These strategies are conventionally called religious and/or spiritual interventions (RSIs). RSIs typically propose the introduction of reflective discussion of R/S values and beliefs into the patient´s treatment as a complementary health therapy. The purpose of RSIs in this context is to improve the spiritual and physical dimension of patients and help them better cope with the disease through religious beliefs and traditions [15, 16] and/or spiritual transcendence [17, 18].

Other authors consider strategies based on ancient practices, such as yoga [19], meditation [20] and tai chi chuan [21] as RSISs. Although theses interventions offer good health outcomes, they involve pre-defined protocols based on specific practices and typically require the patient to follow a particular lifestyle. Previous research has already systematically investigated this type of intervention and is therefore not the focus of the present study.

Despite the clinical possibilities of RSIs, few randomized controlled clinical trials are available in the literature. This methodology offers the most robust scientific evidence to assess the possible mechanisms of action of R/S and to develop the clinical applicability of the discussed constructs [22]. Therefore, the present study aimed to focus on previously published controlled clinical trials to examine whether RSIs can promote healthy behaviors and improve physical health and quality of life. As a secondary goal, this study proposed to identify the types of protocols described and used in the literature, and which populations received the RSIs.

## Methods

A systematic review following the PRISMA guidelines (Preferred Reporting Items for Systematic Reviews and Meta-Analysis) was conducted as described below [23]. The study was conducted between January 2011 and July 2016. Data on mental health outcomes are reported elsewhere [24].

### Selection criteria

Randomized clinical trials that used any RSI protocol for outcomes in physical health or quality of life were included. There were no restrictions with respect to diagnosis or publication date (to July 2016); however, the search was limited to English, Spanish, and Portuguese. Due to the importance of the randomization method in the quality of a clinical trial, this procedure was defined according to the international guidelines for clinical trials methodology proposed by CONSORT (Consolidated Standards of Reporting Trials) [22].

### Search strategies

A Boolean expression was created to search the literature to discover the most relevant articles on the topic: "(spiritu* OR relig* OR faith OR holistic OR multifaith) AND (assistance OR intervention OR treatment OR therapy OR assessment OR group OR meditation) AND (clinical trial OR meta-analysis OR randomized controlled trial OR controlled clinical trial)". Seven different databases were screened: PubMed, Scopus, Web of Science, PsycINFO, The Cochrane Collaboration, Embase and Scielo. For these last two databases, the search terms were adapted to fit their particular search requirements.

### Selection phase

Phase 1: The title and abstract of all papers were reviewed by two independent researchers (CBC and JPBG). Studies were excluded if (a) R/S was not assessed, (b) other methodologies were presented, and (c) repeated versions in databases were identified.

Phase 2: All included articles were fully read to assess: (a) the RSI protocols, (b) the randomization process, and (c) the methodological quality of the articles. The authors of the studies who did not provide full details of randomization were contacted by email, and only those who provided adequate data were included.

### Data items for extraction

The outcomes selected from the final articles included (a) diagnosis of the participants; (b) type of approach used in the RSIs; (c) frequency, duration and follow-up procedures; (d) facilitator of the intervention; and (e) clinical outcomes and their results (physical, preventive, quality of life, spiritual care in practice and satisfaction with the procedure).

### Risk of bias in individual studies

The risk of bias across studies was assessed through the Cochrane Back Review scale, an instrument from The Cochrane Collaboration composed of eleven methodological items that offer an extensive evaluation of a clinical trial design [25]. The score was chosen to provide a better understanding of the items because it was very similar to the CONSORT guidelines for non-pharmacological clinical trials that do not blind patients or providers of interventions. The cut-off was six or more points [26]. Three independent researchers (GL, HV and JPBG) classified the items, and the disagreements were resolved topic by topic by consensus.

### Statistical analysis

Given that the present study is a systematic review, a description of the outcomes found in the selected articles is presented in the tables and figures. Due to the diversity of primary diagnoses and clinical outcomes assessed in the data, it was not possible to perform a meta-analysis. Data extracted in the articles were transformed and presented in Cohen d effect size with 95% confidence interval (CI) when possible using the Campbell Collaboration online calculator [27]. The Cohen-d effect size thresholds used included small (0.20), medium (0.50), large (0.80) and very large (1.30) [28].

To evaluate the reliability of the reviewers in assessing the methodological quality, we used the intraclass correlation coefficient that quantifies the variability among different investigators. The coefficient score ranges from 0 to 1.00: the closer to 1.00. The closer to 10.00, the less variability among the reviewers. For the analysis, the SPSS version 17.0 (SPSS, Inc.) was used.

## Results

### Study selection

**Fig 1:**
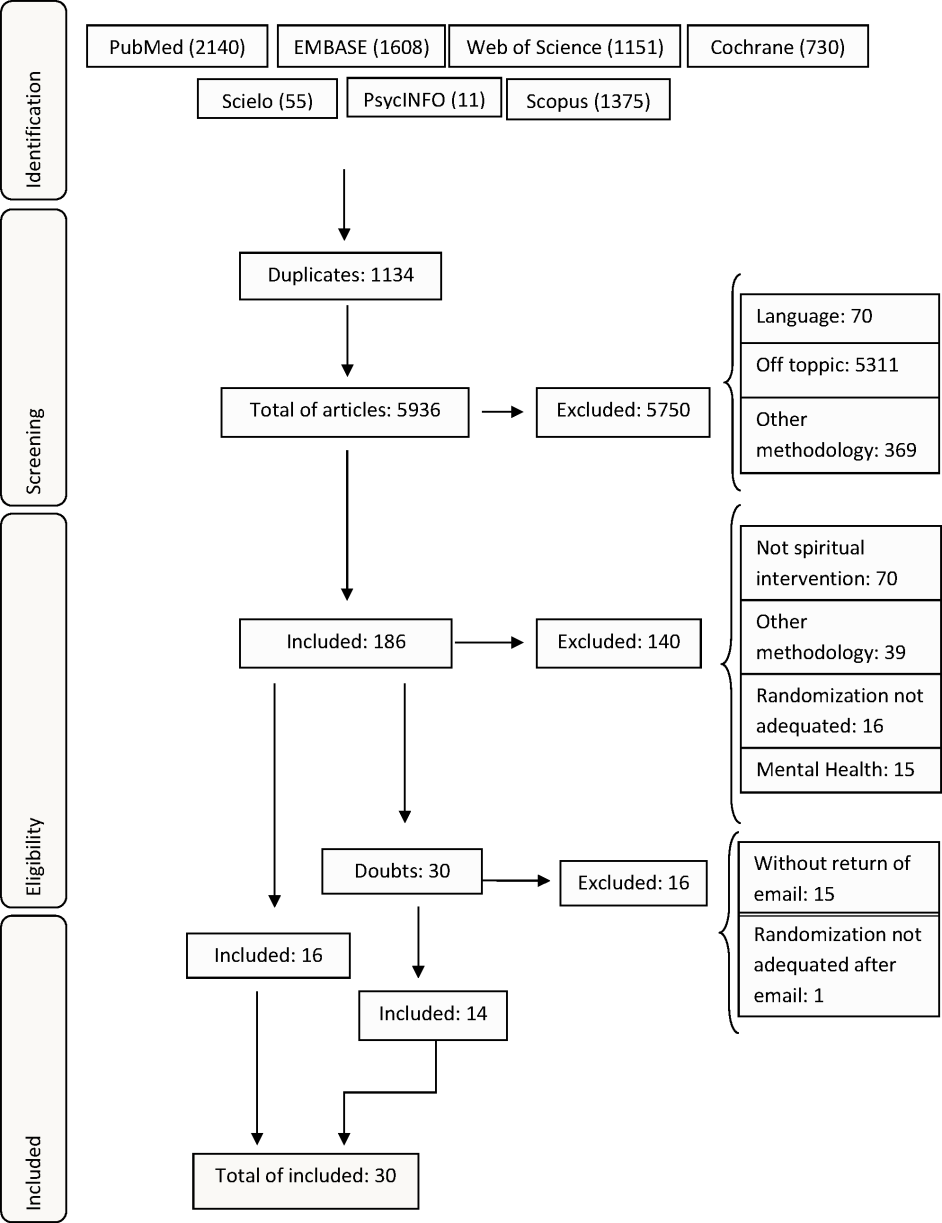
Flowchart of study selection.

Fig 1 presents the flowchart for the selection of the articles in the databases. The search returned a total of 7,070 articles, of which 1,134 were excluded because they were duplicates in different databases. In *Phase* 1, 5,750 articles were eliminated because they were off topic, used other methodologies other than clinical trials, or were in languages not defined in the inclusion criteria. Of the remaining 186 articles examined in *Phase* 2, we excluded 125 articles that were incompatible with the inclusion criteria and 15 that exclusively assessed mental health outcomes. In total, 46 articles remained in this *phase*, including 30 articles that did not elucidate the randomization procedure. The authors of these articles were contacted, and 15 of them did not return the e-mails, and one study used an inadequate process. Thus, 14 studies were included. The final included articles totaled 30.

### Clinical outcomes

The 30 final studies presented very different characteristics. Therefore, we decided to group them into their main research outcomes as follows: a) quality of life, b) weight and physical activity, c) pain, d) other outcomes, e) promotion of healthy behaviors, f) clinical practice of health professionals, and g) satisfaction with protocols proposed.

#### Quality of life– 10 studies

Table 1 presents RSIs proposed to investigate quality of life in different chronic diseases with a total sample of 756 individuals. Patients with cancer (assessed in 4 studies) represented 45.5% of the total sample. Most the interventions (90%) focused on spiritual approaches with only one (10%) using religious aspects to improve quality of life. A predominance of psychotherapy protocols (50%) was noted followed by meditation (40%).

**Table 1:** Characteristics of religious and spiritual interventions in quality of life outcomes.

Author	Population/ Condition	Sample Size	Type of Intervention	Focus of Intervention	Facilitators	Sessions/ Duration (min)	Control Groups	Follow Up (months)	Outcomes and Results Assessed (Cohen d [IC:95%])	Score
Binaei, 2016	Heart failure	46	Psychotherapy	Religious	Author, theologists and psychiatrist	6/ 60	EdCG	1 to 6	QLI Post-treatment: d = 1.18 [0.55; 1.80] QLI 1-month: d = 1.14 [0.52; 1.77]	6
Bormann, 2006	HIV+	93	Meditation	Spiritual	Nurses	5/ 90	EdCG	1 to 6	Q-LES-Q Post-treatment: d = 0.04 [-0.45; 0.36] Q-LES-Q 3-months: d = 0.25 [-0.66; 0.15]	8
Bormann, 2008	Post-traumatic stress disorder	29	Meditation	Spiritual	Authors	6/ 90	WLG	1 to 6	Q-LES-Q:SF: d = 0.70 [-0.05; 1.44]	8
Breitbart, 2012	Cancer	120	Psychotherapy	Spiritual	Clinical psychologist and massage therapist	7/ 60	EdCG	1 to 6	MQOL Post-treatment: d = 0.26 [-0.18; 0.71] MQOL 2-month: d = 0.01 [-0.47; 0.49]	7
Elias, 2015	Breast cancer with mastectomy	28	Guided visualization	Spiritual	Authors	5 to 6/ N/M	TCG	< 1	WHOQOL-B: d = 0.33 [-0.51; 1.18]	5
Jafari, 2013	Cancer	65	Psychotherapy	Spiritual	Spiritual healers	6/ 120–180	EdCG	1 to 6	EORTC QLG: d = 2.17 [1.55; 2.78]	5
Oman, 2006	Healthy	58	Meditation	Spiritual	Authors	5/ 90	WLG	1 to 6	LSI: d = 0.93 [-1.18; 3.05]	7
Piderman, 2013	Cancer	131	Psychotherapy	Spiritual	Health professionals and chaplains	6/ 90	EdCG	1 to 6	LASA: d = 0.48 [-0.13; -0.83]	6
Waccholtz, 2008	Migraine	83	Meditation	Spiritual	Research assistant	30/ 20	TCG	1 to 6	MSQLS: d = 0.46 [-0.15; 1.07]	8
Wu, 2016	Patients with dementia	103	Psychotherapy	Spiritual	Authors	6/ 60	EdCG	< 1	LSI: d = 0.43 [0.04; 0.82]	6

EdCG = Educational Control Group; WLG = Waiting List Group; TCG = Therapeutic Control Group; QLI = Quality of Life Index; Q-LES-Q:SF = Quality of Life Enjoyment and Satisfaction Questionnaire-Short Form; MQOL = McGill Quality of Life Questionnaire; WHOQOL-B = World Health Organization Quality of Life-Brief; EORTC QLG = European Organization for Research and treatment of Cancer Quality of Life; LSI = Life Satisfaction Index; LASA = Linear Analog Self-Assessment; MSQLS = Migraine Specific Quality of life Scale.

Concerning the scales adopted by the authors, only the Life Satisfaction Index was used in two different studies. The Quality of Life Enjoyment and Satisfaction Questionnaire was used in both a full and brief forms in two studies. The mean score of the methodological quality on the Cochrane Scale was 6.6 (SD = 1.17) points. Risk of bias was suspected in two studies in this group (20%).

Concerning quality of life, four of the five studies on the psychotherapy approach presented statistical significance favoring RSIs with different effect sizes: one large (d = 2.17, 95% CI [1.55; 2.78]) [29], two small (d = 0.43, 95% CI [0.04; 0.82]; d = 0.48, 95% CI [-0.13; -0.83]) [30, 31] and one insignificant (d = 0.01, 95% CI [-0.47; 0.49]) [17]. Similarly, two of four studies that used meditation protocols were significant with large (d = 0.93, 95% CI [-1.18; 3.05]) [32] and small (d = 0.25, 95% CI [-0.66; 0.15]) [18] effect sizes.

#### Weight and physical activity– 5 studies

The studies that investigated outcomes related to weight and physical activity assessed 191 individuals from mainly obese and sedentary populations, which represented 74.3% of the total sample (Table 2). The protocols used included church environment (60%) and psychotherapy (40%), and the predominant focus of intervention in these outcomes involved religion in 60% of the articles.

**Table 2:** Characteristics of religious and spiritual interventions in weight and activity outcomes.

Author	Population/Condition	Sample Size	Type of Intervention	Focus of the Intervention	Facilitators	Sessions/Duration (min)	Control Groups	Follow Up (months)	Outcomes and Results Assessed (Cohen d [IC:95%])	Score
Anderson, 2013	Healthy old people	27	Church	Religious	Authors with certification in faith community nursing	10/ 90	EdCG	< 1	Total kcal/day (7-D-PAR): d = 0.80 [-1.59; 0.01] Physical activity (7-D-PAR): d = 0.22 [-0.55; 0.99] Walking (7-D-PAR): d = 0.67 [-0.11; 1.46] Muscle strength (7-D-PAR): d = 1.34 [0.49; 2.19] 6-min walk: d = 0.15 [-0.92; 0.06] SEE: d = 0.28 [-0.49; 1.05] EBBS: d = 0.43 [-0.34; 1.21]	7
Djuric, 2009	Cancer survivors	22	Psychotherapy	Spiritual	Spiritual counselor with master´s degree in psychology and registered dietitian	13/ N/M	TCG	> 6	Health Eating Index (B98FFQ): d = 0.68 [-0.17; 1.54] Physical Activity (B98FFQ): d = 0.17 [-1.00; 0.67] Fruit (B98FFQ): d = 1.83 [0.83; 2.82] Fat calories (B98FFQ): d = 0.49 [-1.34; 0.35] Weight change (kg): d = 0.03 [-0.87; 0.80]	7
Duru, 2010	Sedentary	62	Church	Religious	Research assistants	8/ 90	EdCG	1 to 6	Steps/week: *MD = 7,457 (SD = 3,020) (p = 0.02) Activity in hours/week: *MD = 2.6, (SD = 3.8) (p = 0.50) Weight (kg): *MD = -0.8, (SD = 1.3) (p = 0.51)	8
Fitzgibbon, 2005	Obesity	46	Psychotherapy	Religious	Authors	12/ 90	TCG	1 to 6	BMI: d = 0.03 [-0.54; 0.62] Weight (kg): d = 0.02 [-0.60; 0.56] Dietary fat (7D-PAR): d = 0.11 [-0.49; 0.70] Energy expenditure (7D-PAR): d = 0.16 [-0.76; 0.43] Moderate activity (7D-PAR): d = 0.19 [-0.79; 0.41]	7
Krukowski, 2010	Obesity	34	Church	Religious	Graduate student with previous knowledge of topic	16/ 60	EdCG	1 to 6	Weight loos (kg): d = 0.10 [0.57; 0.77] Weight regain (%) 6-months: d = 0.36 [-1.06; 0.34]	6

EdCG = Educational Control Group; TCG = Therapeutic Control Group; 7-D-PAR = The Stanford Seven-Day Physical Activity Recall; SEE = Self-Efficacy for Exercise Scale; EBBS = Exercise Benefits and Barriers Scale; B98FFQ = Block ´98 Food Frequency Questionnaire; BMI = Body Mass Index.

*MD = Mean Difference (SD = Standard Deviation), article without data to calculate Cohen d.

All studies used control groups (with another type of activity), and the mean score of the Cochrane Scale was 7 (SD = 0.71) points. The RSI protocols concerning loss of weight exhibited insignificant effect sizes in three of the four studies [33–35]. In addition, RSIs on physical activity exhibited a small effect size (d = 0.22, 95% CI [-0.55; 0.99]) [36] and two insignificant results [34, 35]. Finally, RSIs exhibited an insignificant effect size when assessing eating habits (d = 0.11, 95% CI [-0.49; 0.70]) in one of the three studies evaluated [33].

#### Pain– 5 studies

Studies on pain assessed individuals with chronic disease (63.7%) and those in a healthy population (36.3%) in a total sample of 403 (Table 3). The protocols were mostly spiritual (80%) and based on meditation approaches (60%), and all protocols were facilitated by the authors. Meditation protocols compared the intervention with therapeutic approaches in the control groups, whereas the others used educational approaches. The Cochrane scale revealed a mean of 8 points (SD = 0.71).

**Table 3:** Characteristics of religious and spiritual interventions in pain outcomes.

Author	Population/Condition	Sample Size	Type of Intervention	Focus of Intervention	Facilitators	Sessions/Duration (min)	Control Groups	Follow Up (months)	Outcomes and Results Assessed (Cohen d [IC:95%])	Score
Duru, 2010	Sedentary	62	Church	Religious	Research assistants	8/ 90	EdCG	1 to 6	AGSPS: *MD = 0.6, (SD = 3.5) (p = 0.87)	8
Feuille, 2013	Migraine	74	Meditation	Spiritual	Research assistant	15/ 20	TCG	< 1	Severity of pain (VAS): d = 0.05 [-0.59; 0.49] Pain tolerance (CPT): d = 0.12 [-0.42; 0.66]	7
McCauley, 2011	Chronic pain	100	Audiovisual	Spiritual	Material made by authors	5/ 28	EdCG	1 to 6	Pain (VAS): d = 0.06 [-0.48; 0.36]	9
Wachholtz, 2005	Healthy	84	Meditation	Spiritual	Authors	14/ 20	TCG	1 to 6	Pain tolerance (CPT): d = 0.27 [-0.85; 0.30]	8
Wachholtz, 2008	Migraine	83	Meditation	Spiritual	Research assistant	30/ 20	TCG	1 to 6	Frequency of pain (monthly): d = 0.26 [-0.86; 0.35] Severity of pain (VAS): d = 0.11 [-0.72; 0.49] Pain tolerance (CPT): d = 0.63 [0.01; 1.25]	8

EdCG = Educational Control Group; TCG = Therapeutic Control Group; AGSPS = American Geriatric Society Pain Score; M = Mean; SD = Standard deviation; VAS = Visual Analog Scale; CPT = Cold Pressor Task.

*MD = Mean Difference, article without data to calculate Cohen d.

RSI assessed pain tolerance using the same method in three articles. Two studies exhibited a small effect size (d = 0.12, 95% CI [-0.42; 0.66]; d = 0.27, 95% CI [-0.85; 0.30]) [37, 38], and the third study was not significant [39]. Concerning the frequency of pain measured in two studies, RSI exhibited one small effect size (d = 0.26, 95% CI [-0.86; 0.35]) [39] and a very small effect size [40]. Severity of pain was also assessed in the two articles, and the RSIs assessed exhibited very small effect sizes in both [37, 39].

#### Other outcomes– 5 studies

In S1 Table, we present other physical outcomes investigated with RSIs in individuals with chronic diseases. No pattern was noted regarding the type or focus of intervention. Facilitators were mainly the authors of the studies (80%).

RSI on levels of salivary cortisol exhibited a small effect size (d = 0.28, 95% CI [-0.75; 0.19]) measured post-treatment that was not maintained in follow up [41]. Other outcomes assessed in the RSI, including daily activity (d = 0.09, 95% CI [-0.43; 0.26]), management of chronic disease (d = 0.19, 95% CI [-0.24; 0.61]) and energy, exhibited insignificant effect sizes (d = 0.18, 95% CI [-0.24; 0.61]). Concerning cardiac measures, the RSI was not statistically significant [42].

#### Promotion of healthy behaviors– 5 studies

Some studies were designed to stimulate healthy behaviors (S2 Table). Three articles evaluated RSIs in 849 health individuals in church environments (81.7% of the total sample), and two studies assessed chronic conditions of health in 190 individuals through psychotherapy models (18.3%). Most control groups (80%) used educational approaches to compare the RSIs, and the mean score on Cochrane scale was of 6.2 points (SD = 1.10).

RSIs on organ donations outcomes exhibited small to medium effect sizes (d = 0.33, 95% CI [0.14; 0.53]; d = 0.63, 95% CI [0.42; 0.82]) favoring RSIs [43]. Concerning cancer screening, RSIs showed small effect sizes in the ideas about health impacts (d = 0.22, 95% CI [-0.15; 0.04]) [15], on the benefits of cancer screening (d = 0.22, 95% CI [-0.05; 0.48]) and on the benefits of colonoscopy (d = 0.19, 95% CI [-0.07; 0.46] [16]. Other measures of cancer as barriers and benefits of screening tests were insignificant in RSIs.

The prevention of HIV by sexual transmission revealed no differences between the groups assessed [44], but the measures of the risk behavior exhibited significant differences in the RSI protocol [45].

#### Clinical practice of healthcare professionals– 4 studies

There were four studies that investigated the impact of the RSIs on the clinical practice of 216 health professionals (S3 Table). Half of the studies used psychotherapy approaches, and the other half used meditation protocols. However, all of them focused on spiritual models facilitated by the authors of the studies. Only one used an educational protocol as a control group, whereas the other three studies compared RSIs with a waiting list group. The mean score on Cochrane scale was 6.25 points (SD = 0.96).

Eleven different measures regarding burnout, spiritual care and spiritual practice of the professional were assessed in the four studies and only the personal accomplishment exhibited no significance between the groups [32]. Effect sizes of RSIs evidenced a large effect on emotional exhaustion (d = 1.0, 95% CI [-4.33; 2.33]) [32], small effect on relational self-assessed caregiving (d = 0.40, 95% CI [0.08; 0.72]) [46] and an insignificant effect on job satisfaction (d = 0.08, 95% CI [-0.44; 0.60]) [32].

#### Satisfaction with the proposed protocols– 3 studies

Some authors also investigated the satisfaction of individuals with RSIs protocols. Patients with generalized anxiety disorder received religious intervention by audiovisual resources, and the Client Satisfaction Questionnaire revealed no significant difference (d = 0.90, [0.42; 1.35]) [47]. Another study assessed this measure in patients with post-traumatic stress disorder after spiritual mediation, and the authors reported moderate to high approval of the protocol by 86% of participants [48]. The latter study evaluated spiritual psychotherapy in drug users, and the client´s credibility of the protocol exhibited a mean of 3.44 (SD = 0.42) on a scale of 0 to 4. Client´s satisfaction exhibited a mean of 8.85 (SD = 1.15) on a scale of 0 to 10 [44].

### Intervention protocols

Regarding the protocols used, we noted a basic distinction of RSIs into two models: spiritual and religious. The spiritual models focused on moral/spiritual values and coping with the disease through transcendence and personal beliefs. Authors claimed that this approach can include atheists and agnostics due to the general approach of the beliefs discussed. The religious models used the traditions of religious beliefs, such as those of Catholicism or Judaism, to help people who already identified themselves as belonging to a specific religious tradition. Of the 30 articles included, the approaches were distributed in 19 (63.3%) spiritual and 11 (36.7%) religious models. Regardless of the religious or spiritual focus, different protocols were proposed for each model that shared some features as presented below.

#### Psychotherapy– 12 studies

The authors who used psychotherapy to promote RSIs introduced religious/spiritual values and beliefs into the program to help cope with the diseases. Ideas, such as “God is a source of hope, forgiveness, peace and acceptance”, “Higher Power gives relief and meaning in the end-of-life” or “feel the connection with others through love”, were used as topics. Some authors also provided practical activities, i.e., as prayers and reading of sacred texts in groups, discussion to promote transcendence and changes in unhealthy behaviors and habits.

Therapeutic [17, 30, 31, 34, 35, 44, 49] or educational approaches [29, 45, 50–52] were used. Control groups included traditional psychotherapy [34, 35, 44, 49], educational models [17, 29–31, 45, 50, 52] and waiting list [51]. Only one study offered an individual protocol [17], and the remaining proposals used group discussion.

#### Meditation– 8 studies

The R/S meditations consisted of phrases about God or a Higher Power being chosen and mentally repeated by patients. Stories about spiritual leaders were also supplied by some authors to be as examples of attitudes. Control groups in this type of intervention used traditional meditation methods [37–39], waiting list [32, 46, 48] and informational videos about the disease [18, 41]. Facilitators met with participants before and/or during the study period to eliminate doubts about the protocol.

#### Church approaches– 6 studies

Church approaches were typically performed by religious ministers (priests, clerics and pastors) and occasionally by the authors of the study concomitantly. Reading sacred texts, praying in groups, discussing and demystifying polemic subjects with the religious leaders (i.e. sex, organ donation) were some of the teachings incorporated into RSI to promote valuing life and responsibility with the body.

The material used in church interventions was developed by the corresponding authors of the studies in collaboration with the religious ministers [15, 16, 33, 36, 53]. The authors trained the leaders prior to the procedures. As a comparison group, regular protocols performed by institutions that focused on health promotion and diseases prevention were used.

#### Audiovisual resources– 2 studies

In this type of intervention, both studies created videos that were available for patients to watch on their own. The contents included religious/spiritual themes to cope with stress, to change unhealthy habits and to inspire gratitude and forgiveness. Patients were asked to write down reflective notes after watching the videos. One study compared the RSIs with a video showing muscular exercises and a waiting list group [47], whereas the other study exclusively used individuals who viewed an educational video about the disease as the control group [40].

#### Guided visualization– 2 studies

The guided visualization studies used exercises concerning visualization of divine/spiritual beings helping and accompanying the patient’s life. Images of light surrounding the ambient and love connecting the person with the rehabilitation were verbally conducted by the authors. The RSIs were compared with brief psychotherapy [54] and respiratory relaxation [42].

Another point regarding the facilitators of the interventions should be made. Some of the facilitators had previous knowledge of the technique that was used in the intervention and experience of working with R/S topics [29, 30, 32, 40, 46, 49, 52–54]. For those who did not, training was both offered to health professionals when they were not familiar with the subject of the R/S [33, 39, 43, 47] and to the religious ministers about the technique´s approach [15, 16, 35, 45]. Each intervention contained a program for the adequate training for the proper use of the process, and the quality of the content offered was thoroughly assessed regardless of the type of protocols used.

### Methodological quality of the articles

Concerning the reliability between examiners, the intraclass correlation coefficient demonstrated positive reliability in the interpretation of the scales (0.832, 95% confidence interval: 0.752 to 0.893).

Table 4 presents the items assessed on the Cochrane scale. The final score ranged from five to nine points, with an average of 6.83 (SD = 9.08). In total, 86.7% of studies were above the cut-off point, and 30% had a score of eight or nine. We observed that none of the research fulfilled the fields for patient and facilitator blinding (D and E), which prevented the articles from achieving the maximum score. The blinding of the outcome assessor (F) was present in 6 studies but not mentioned in 11 studies. Despite the use of different protocols, the intensity, duration and frequency of the procedures (H and J) were reported in 73.3% of the articles. Risk of bias was suspected in 13.3% [29, 45, 52, 54] of the studies based on the final scores received.

**Table 4:** Assessment of the methodological items evaluated on Cochrane classification.

Author	A	B	C	D	E	F	G	H	I	J	K	Score
Anderson, 2013	+	+	+	-	-	-	+	-	+	+	+	7
Arriola, 2010	+	+	+	-	-	-	+	+	-	+	-	6
Binaei, 2016	+	+	+	-	-	NM	+	+	-	+	NM	6
Bormann, 2006	+	+	+	-	-	-	+	+	+	+	+	8
Bormann, 2008	+	+	+	-	-	+	+	-	+	+	+	8
Bormann, 2009	+	+	+	-	-	-	+	+	+	+	+	8
Breitbart, 2012	+	+	NM	-	-	NM	+	+	+	+	+	7
Burkhart, 2012	+	-	+	-	-	NM	+	+	NM	+	NM	5
Djuric, 2009	+	+	+	-	-	NM	+	+	+	+	-	7
Duru, 2010	+	+	+	-	-	+	+	+	-	+	+	8
Elias, 2015	+	-	+	-	-	NM	+	+	-	+	NM	5
Feuille, 2013	+	+	+	-	-	+	-	+	+	+	-	7
Fitzgiboon, 2005	+	+	+	-	-	-	+	+	+	+	-	7
Guilherme, 2016	+	+	NM	-	-	-	+	+	+	+	+	7
Holt, 2008	+	+	+	-	-	+	+	+	+	+	-	8
Holt, 2012	+	+	-	-	-	NM	+	+	+	+	-	6
Jafari, 2013	+	NM	+	-	-	-	+	-	+	+	NM	5
Koenig, 2015	+	+	+	-	-	+	+	+	+	+	+	9
Krukowski, 2010	+	+	-	-	-	NM	+	+	+	+	NM	6
Margolin, 2006	+	+	+	-	-	-	+	+	+	-	-	6
Mccauley, 2011	+	+	+	-	-	+	+	+	+	+	+	9
Morita, 2009	+	+	+	-	-	-	+	-	+	+	-	6
Oman, 2006	+	+	+	-	-	-	+	-	+	+	+	7
Oman, 2008	+	+	+	-	-	-	+	-	+	+	+	7
Piderman, 2013	+	+	+	-	-	-	-	+	+	+	-	6
Rosmarin, 2010	+	+	+	-	-	NM	+	+	-	+	+	7
Wachholtz, 2005	+	+	+	-	-	NM	+	+	+	+	+	8
Wachholtz, 2008	+	+	+	-	-	NM	+	+	+	+	+	8
Wingood, 2013	+	NM	-	-	-	NM	-	+	+	+	+	5
Wu, 2016	+	+	+	-	-	-	+	-	+	+	-	6

A = randomization method/B = allocation concealed/C = similar baseline/D = patient blinded/E = provider blinded/F = assessor blinded/G = cointervention avoided/H = acceptable compliance/I = acceptable drop out/J = timing of outcome of assessment similar/K = intention to treat analysis

## Discussion

Due to the growing interest in the effects of R/S as a complementary treatment in health care, we conducted a systematic review of randomized controlled trials without delimitation of time of publication. The studies revealed a low risk of bias with an average of 6.83 points in the Cochrane scale, which is greater than the cut-off of 6 points. Although diverse populations were assessed in the articles, the main outcomes investigated were grouped into seven items. The RSIs on quality of life, physical activity, pain, promotion of health behaviors and clinical practice of health professionals tended to exhibit small effect sizes favoring the RSIs compared with other complementary strategies used as control groups. Other outcomes, such as cardiac measures, and satisfaction with the protocols, showed no consensus, due to the absence of similar instruments of assessment of the outcomes among the studies. The five different types of intervention–psychotherapy, meditation, church approaches, audiovisual resources and guided visualization–adopted either a religious or spiritual approach, and the most commonly used approach was psychotherapy in 40% of the articles.

Indeed, a significant increase in the search for different types of complementary therapies has occurred in recent decades. A previous systematic review revealed an increase of 9% to 65% in the prevalence of the use of complementary therapy in health care between 1988 and 1997 [55]. These therapies have demonstrated promising results when combined with conventional treatment, including a reduction of physical symptoms and an increase in quality of life, especially in chronic conditions [56] and with terminally ill patients [57]. Treatments, such as herbal therapies, relaxation techniques, chiropractic, acupuncture, meditation and yoga, are very popular among patients with chronic diseases [58]. Similarly, Barnes et al. [59] demonstrated that praying for oneself and for others is the complementary therapy most used by Americans.

RSIs applied to mental health are more common than those applied for physical outcomes. Therefore, the similarities in the validated scales and measures for the symptoms provided a few meta-analyses [24, 60–62]. Those published more recently considered the methodological quality in the processes of the systematic review. Oh and Kim revealed a moderate effect size for depression and anxiety (d = 0.69, 95% CI [0.05; 1.34]) [62], and Gonçalves et al. presented a moderate effect (d = 0.43, 95% CI [0.25; 0.61]) for anxiety, but insignificant effect (d = 0.11, 95% CI [0.03; 0.25]) for depression [24].

Kruizinga et al. [63] assessed quality of life in patients with cancer and found that RSIs had a moderate effect size (d = 0.50, 95% CI [0.20; 0.79]) after 2 weeks of treatment. However, the result was insignificant (d = 0.14, 95% CI [0.05; 0.33]) when the articles met the criteria of allocation concealment. In contrast to our study, they considered practices, such as yoga, meditation and tai chi chuan, as RSIs. Given the particularities and the long history of these approaches as complementary therapies [19–21], we did not include them in our study. The final 10 articles that assessed RSIs on quality of life in our review used different scales to measure it. Therefore, we opted not to perform a meta-analysis [64]. Four of the studies revealed no significant differences between the groups. Two studies demonstrated large effect size of RSIs, however it should be interpreted more carefully given that one study had a low score on Cochrane scale and the other study used a waiting list as the control group. Of the remaining four studies, three showed a small effect size, and the remaining study revealed a very small effect favoring RSIs. Our findings are consistent with the aforementioned meta-analyses in which RSIs with better methodological quality exhibit small effects on quality of life when compared with other complementary therapies.

To investigate RSIs in relation to biological outcomes, Oh and Kim [62] performed a meta-analysis with different diagnoses and observed a small effect size favoring RSIs regarding pain (d = 0.39, 95% CI [0.55; 0.23]). The heterogeneity between the articles was high (I^2^ = 72%), likely because the study included non-randomized controlled trials. Our five final studies that investigated pain presented the best average on the Cochrane scale (8 points). Although these studies assessed pain using different concepts and methods, similarities were noted concerning the type and focus of interventions. A small effect size favoring RSIs was noted, based on the trustworthy nature of the methodological quality of the articles.

Hulett and Armer [65] also assessed psychoneuroimmunological outcomes in survivors of cancer and argued that RSIs improve or stabilize the outcomes despite their designs limitations. In our review, studies that investigated cortisol, daily activity and management of chronic diseases exhibited very small effect sizes. Cardiac outcomes, however, did not differ between the groups assessed. Biological outcomes should be further investigated before any conclusions regarding the efficacy of the RSIs are drawn.

We identified additional outcomes that were investigated from the perspective of the RSIs proposals that were not mentioned in previous systematic reviews in the literature. Similar types and focuses of intervention were employed for the loos of weight/physical activity and promotion of health behaviors, and good methodological quality analyses were performed. However, very different scales and outcomes were assessed. Nevertheless, the majority of the results revealed very small results favoring RSIs compared with the control groups proposed (either educational and therapeutic approaches). RSIs do not better influence these outcomes compared with other complementary therapies proposed. Finally, regarding RSIs in the clinical practice of healthcare professionals, the results of the studies were imprecise. No conclusions about the efficacy of RSIs on these types of outcomes could be drawn.

The evidences of the efficacy of RSIs in health identified in our study does not support a high quality of clinical recommendation. Specific recommendations concerning an action would be beyond the scope of a systematic review and should be made by clinical practice guideline developers [66]. The choice of RSIs to the detriment of others should be considered carefully and made in agreement between the health professional and the patient, and even include the family, if necessary. Many patients claim a need to discuss their R/S beliefs during health treatments [2, 67], and some health professionals feel motivated when verify clinical improvements in their patients are verified after approaching the subject [14, 51]. However, caution is recommended when using these treatments, as this is a private and personal matter that should be considered but not used as a prescription [68]. In addition, some doctors do not feel prepared to integrate R/S in clinical practice [69–71]. These observations endorse the necessity of introducing the R/S topic into medical schools to clarify the clinical implications and professional conduct in the field [72, 73].

In reference to the types of interventions, the R/S topic was explored using different methods according to the objective of the intervention. Some were developed by the authors to include any or no religious affiliation, and others were related to a specific religious tradition. The main theme also seemed to be flexible and involve different complementary therapy techniques. The notions of sacredness and the transcendence were stimulated in patients and healthy people through different pathways, such as discursive talk, dynamic group, individual meditation or audiovisual reflections.

Another important point to note in the clinical trials was the exchange of knowledge between the research team and the facilitators of the RSIs. Proper preparation to conduct the RSIs also seemed to be an important factor in avoiding an impression of a punitive approach that religion can occasionally suggests. In cases where a condition is viewed as a “divine punishment”, levels of anxiety, depression and even mortality can increase [74]. If the approach is misinterpreted by patients, the outcomes can provide grief rather than relief [75, 76]. Thus, it is notable that the facilitators ensured that they generally produced the intended positive effect in the patients.

Finally, regarding to the methodological quality of RSIs, we emphasize the importance of minimizing bias in these studies. Due to the absence of double blinding in the studies, meaning that both patient and facilitator are aware of the type of intervention that is being offered, clinical trials in this field have limitations in their performance. With this in mind, it is fundamental to follow the CONSORT guidelines. In 2008, was published an extension to the CONSORT statement that focused on non-pharmacological research and highlighted the relevance of some methodological items that could make a difference in the quality of these studies [77]. These items included the randomization sequence and allocation of participants. It was suggested that these tasks be performed by a person who has no influence on the eligibility of the patients. A second item covered the impartiality of an assessor for the outcomes to avoid biases during final data assessment [23]. Of the final thirty studies considered in our review, only 4 described the use of this strategy, whereas 11 do not mention it. These items do not demand too much additional effort and are attainable for RSIs. Thus, these items should be considered. The similarity in the protocols regarding the experimental and control groups is also another point that should be noted [22]. With respect to the frequency, duration and follow-up of interventions, authors should try to develop standard procedures. Giving proper attention to the methodological design for RSIs can help to produce more solid evidence regarding the effects of this complementary therapy on health.

## Directions for future research

Further investigation in this area is imperative to better understand the mechanisms of action behind RSIs. Although the literature revealed evidence of the benefits of R/S on health, the number and quality of these studies are limited. Knowledge about the mechanisms of action of the RSIs is lacking. The consequences of better understanding will greatly impact in clinical practice.

**Fig 2:**
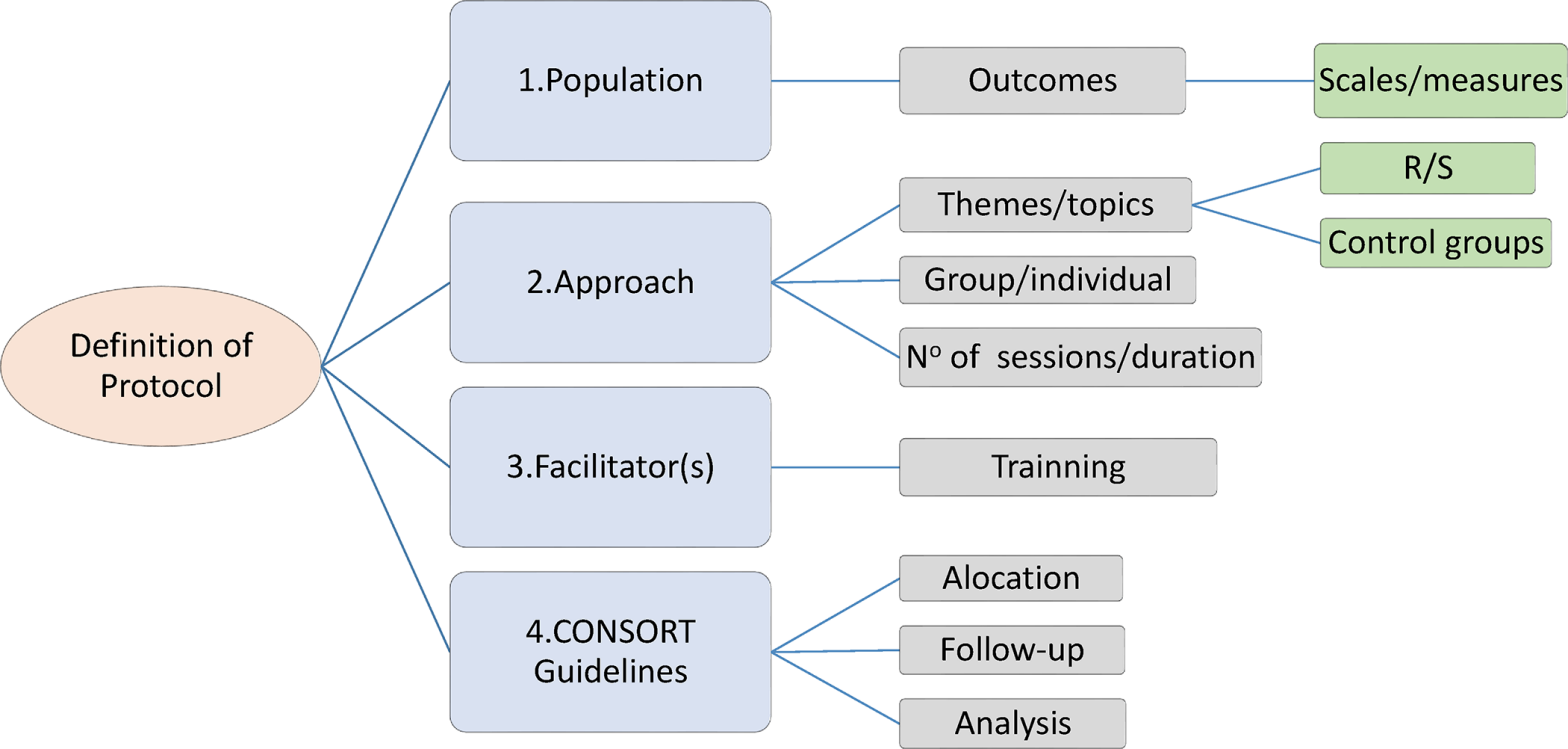
Flowchart of guidelines to create a religious/spiritual intervention.

To contribute to future research in this area, we have suggested a standardized RSI protocol described in a brief flowchart for the conceptualization and construction of this type of intervention (Fig 2). In item 1 (Population), we propose the independent use of religious and spiritual measures into the same research, aiming to understand the possible relationship of the different constructs on the outcome assessed. Item 2 (Approach) seeks to reproduce previously published protocols. We suggest using the same structure in new research, allowing a deeper understanding of the effect of R/S on health. Actual protocols are very divergent, and it is difficult to compare studies. This practice is very important and should always be performed. In item 3 (Facilitators), we recommend exploring the exchange between the research team and the religious/spiritual counselors to guarantee a better instrument of intervention. It would also be interesting if studies could investigate the impact of different facilitators on the planned outcomes. We would also like to highlight the importance of following item 4 (CONSORT), which recommends using these guidelines when preparing and standardizing the content and the training of the facilitators.

## Limitations

Similar to other systematic reviews, it is possible that publications that were not indexed in the databases or written in other languages were not included in this study. However, the medical databases employed and the languages chosen are widely used by the scientific community, covering most of the published studies. The inability to perform a meta-analysis was due to the heterogeneous protocols and outcomes reported in each selected study. Another limitation involves the exclusion of studies that investigated ancient practices and philosophies, due to the specific characteristics of their approach, which follow a pre-determined concept and were previously discussed elsewhere [19–21].

## Conclusion

Compared with other complementary health therapies, systematically selected clinical trials of RSIs have demonstrated small benefits concerning improvements in quality of life and reducing pain, and similar results on weight loss and health behavior promotion were noted. The diversity of approaches in this field indicate a need for more studies using comparable methodologies to understand the mechanisms of action of RSIs and their role as complementary treatments in health care.

## Supporting information

S1 File PRISMA 2009-checklist. Checklist of the items of the article following PRISMA guidelines (Preferred Reporting Items for Systematic Reviews and Meta-Analysis).
(DOC)

S2 File List of excluded articles. List of the excluded articles in the selection articles process.
(DOCX)

S3 File Search strategy. Boolean expression with the key words used for the search of the articles are presented for each database used.
(DOCX)

S1 Table Supplementary material. (DOCX)

S2 Table Supplementary material. (DOCX)

S3 Table Supplementary material. (DOCX)
