# Supplementary material for: Complementary religious and spiritual interventions in physical health and quality of life: A systematic review of randomized controlled clinical trials
Source: PLoS One. 2017 Oct 19;12(10):e0186539. doi: 10.1371/journal.pone.0186539 (PMC5648186; doi:10.1371/journal.pone.0186539)
Supplement: S2 File — List of the excluded articles in the selection articles process. (DOCX) [file pone.0186539.s002.docx]

**Total of excluded: 156**

**Intervention not religious or spiritual: 70**

Abbot, N. C., Harkness, E. F., Stevinson, C., Marshall, F. P., Conn, D. A., & Ernst, E. (2001). Spiritual healing as a therapy for chronic pain: a randomized, clinical trial. *Pain*, *91*(1), 79-89.

Amaro, H., Magno-Gatmaytan, C., Meléndez, M., Cortés, D. E., Arevalo, S., & Margolin, A. (2010). Addiction treatment intervention: An uncontrolled prospective pilot study of spiritual self-schema therapy with Latina women. *Substance Abuse*, *31*(2), 117-125.

Ando, M., Morita, T., Akechi, T., Okamoto, T., & Japanese Task Force for Spiritual Care. (2010). Efficacy of short-term life-review interviews on the spiritual well-being of terminally ill cancer patients. *Journal of pain and symptom management*, *39*(6), 993-1002.

Astin, J. A., Stone, J., Abrams, D. I., & Moore, D. H. (2006). The efficacy of distant healing for human immunodeficiency virus-results of a randomized trial. *Alternative therapies in health and medicine*, *12*(6), 36.

Baig, A. A., Mangione, C. M., Sorrell-Thompson, A. L., & Miranda, J. M. (2010). A randomized community-based intervention trial comparing faith community nurse referrals to telephone-assisted physician appointments for health fair participants with elevated blood pressure. *Journal of general internal medicine*, *25*(7), 701-709.

Beiranvand, S., Noparast, M., Eslamizade, N., & Saeedikia, S. (2014). The effects of religion and spirituality on postoperative pain, hemodynamic functioning and anxiety after cesarean section. *Acta Medica Iranica*, *52*(12), 909-915.

Benson, H., Dusek, J. A., Sherwood, J. B., Lam, P., Bethea, C. F., Carpenter, W., ... & Drumel, D. (2006). Study of the Therapeutic Effects of Intercessory Prayer (STEP) in cardiac bypass patients: a multicenter randomized trial of uncertainty and certainty of receiving intercessory prayer. *American heart journal*, *151*(4), 934-942.

Bock, B. C., Morrow, K. M., Becker, B. M., Williams, D. M., Tremont, G., Gaskins, R. B., ... & Marcus, B. H. (2010). Yoga as a complementary treatment for smoking cessation: rationale, study design and participant characteristics of the Quitting-in-Balance study. *BMC complementary and alternative medicine*, *10*(1), 14.

Boelens, P. A., Reeves, R. R., Replogle, W. H., & Koenig, H. G. (2009). A randomized trial of the effect of prayer on depression and anxiety. *The International Journal of Psychiatry in Medicine*, *39*(4), 377-392.

Brooks, A. J., Schwartz, G. E., Reece, K., & Nangle, G. (2006). The effect of Johrei healing on substance abuse recovery: a pilot study. *Journal of Alternative & Complementary Medicine*, *12*(7), 625-631.

Cannister, M. W. (1999). Mentoring and the spiritual well-being of late adolescents. *Adolescence*, *34*(136), 769-780.

Carson, J. W., Keefe, F. J., Lynch, T. R., Carson, K. M., Goli, V., Fras, A. M., & Thorp, S. R. (2005). Loving-kindness meditation for chronic low back pain results from a pilot trial. *Journal of Holistic Nursing*, *23*(3), 287-304.

Cha, K. Y., Wirth, D. P., & Lobo, R. A. (2003). Does Prayer Influence the Success of in Vitro Fertilization-embryo Transfer? Report of a Masked, Randomized Trial. *Year Book of Psychiatry & Applied Mental Health*, *2003*(1), 265-266.

Chan, C. H., Ng, E. H., Chan, C. L., & Chan, T. H. (2006). Effectiveness of psychosocial group intervention for reducing anxiety in women undergoing in vitro fertilization: a randomized controlled study. *Fertility and sterility*, *85*(2), 339-346.

Chan, C. L., Ho, R. T., Lee, P. W., Cheng, J. Y., Leung, P. P., Foo, W., ... & Spiegel, D. (2006). A randomized controlled trial of psychosocial interventions using the psychophysiological framework for Chinese breast cancer patients. *Journal of Psychosocial Oncology*, *24*(1), 3-26.

Chen, H., Cheal, K., McDonel Herr, E. C., Zubritsky, C., & Levkoff, S. E. (2007). Religious participation as a predictor of mental health status and treatment outcomes in older persons. *International Journal of Geriatric Psychiatry*, *22*(2), 144-153.

Chen, Y. Y. (2005). Written emotional expression and religion: effects on PTSD symptoms. *The International Journal of Psychiatry in Medicine*, *35*(3), 273-286.

Chukumnerd, P., Hatthakit, U., & Chuaprapaisilp, A. (2011). The experience of persons with allergic respiratory symptoms: practicing yoga as a self-healing modality. *Holistic nursing practice*, *25*(2), 63-70.

Cohen, L., Fouladi, R. T., & Katz, J. (2005). Preoperative coping strategies and distress predict postoperative pain and morphine consumption in women undergoing abdominal gynecologic surgery. *Journal of psychosomatic research*, *58*(2), 201-209.

Conner, B. T., Anglin, M. D., Annon, J., & Longshore, D. (2009). Effect of religiosity and spirituality on drug treatment outcomes. *The journal of behavioral health services & research*, *36*(2), 189-198.

Danhauer, S. C., Mihalko, S. L., Russell, G. B., Campbell, C. R., Felder, L., Daley, K., & Levine, E. A. (2009). Restorative yoga for women with breast cancer: findings from a randomized pilot study. *Psycho‐Oncology*, *18*(4), 360-368.

Derose, K. P., Hawes-Dawson, J., Fox, S. A., Maldonado, N., Tatum, A., & Kington, R. (2000). Dealing with diversity: recruiting churches and women for a randomized trial of mammography promotion. *Health Education & Behavior*, *27*(5), 632-648.

Dixon, M. (1998). Does ‘healing’benefit patients with chronic symptoms? A quasi-randomized trial in general practice. *Journal of the Royal Society of Medicine*, *91*(4), 183-188.

Dowd, T., & Kolcaba, K. (2006). Two interventions to relieve stress in college students. *Beginnings (American Holistic Nurses' Association)*, *27*(1), 10-11.

Duan, N., Fox, S. A., Derose, K. P., & Carson, S. (2000). Maintaining mammography adherence through telephone counseling in a church-based trial. *American Journal of Public Health*, *90*(9), 1468.

Engelhardt, J. B., Rizzo, V. M., Della Penna, R. D., Feigenbaum, P. A., Kirkland, K. A., Nicholson, J. S., ... & Tobin, D. R. (2009). Effectiveness of care coordination and health counseling in advancing illness. *The American journal of managed care*, *15*(11), 817-825.

Evans, S., Cousins, L., Tsao, J. C., Sternlieb, B., & Zeltzer, L. K. (2011). Protocol for a randomized controlled study of Iyengar yoga for youth with irritable bowel syndrome. *Trials*, *12*(1), 15.

Finocchario-Kessler, S., Catley, D., Berkley-Patton, J., Gerkovich, M., Williams, K., Banderas, J., & Goggin, K. (2011). Baseline predictors of ninety percent or higher antiretroviral therapy adherence in a diverse urban sample: the role of patient autonomy and fatalistic religious beliefs. *AIDS Patient Care and STDs*, *25*(2), 103-111.

Fitchett, G., & Powell, L. H. (2009). Daily spiritual experiences, systolic blood pressure, and hypertension among midlife women in SWAN. *Annals of Behavioral Medicine*, *37*(3), 257-267.

Fry, R. B., & Prentice-Dunn, S. (2005). Effects of coping information and value affirmation on responses to a perceived health threat. *Health Communication*, *17*(2), 133-147.

Gallez, P. L., Dimmock, S., & Bird, H. A. (2000). Spiritual healing as adjunct therapy for rheumatoid arthritis. *British Journal of Nursing*, *9*(11), 695-700.

Gerard, S., Smith, B. H., & Simpson, J. A. (2003). A randomized controlled trial of spiritual healing in restricted neck movement. *The Journal of Alternative & Complementary Medicine*, *9*(4), 467-477.

Hannon, P. A., Bowen, D. J., Christensen, C. L., & Kuniyuki, A. (2008). Disseminating a successful dietary intervention to faith communities: feasibility of using staff contact and encouragement to increase uptake. *Journal of nutrition education and behavior*, *40*(3), 175-180.

Harris, W. S., Gowda, M., Kolb, J. W., Strychacz, C. P., Vacek, J. L., Jones, P. G., ... & McCallister, B. D. (1999). A randomized, controlled trial of the effects of remote, intercessory prayer on outcomes in patients admitted to the coronary care unit. *Archives of Internal medicine*, *159*(19), 2273-2278.

Henry, M., Cohen, S. R., Lee, V., Sauthier, P., Provencher, D., Drouin, P., ... & Gilbert, L. (2010). The Meaning‐Making intervention (MMi) appears to increase meaning in life in advanced ovarian cancer: A randomized controlled pilot study. *Psycho‐Oncology*, *19*(12), 1340-1347.

Hoover, D. R., & Margolick, J. B. (2000). Questions on the design and findings of a randomized, controlled trial of the effects of remote, intercessory prayer on outcomes in patients admitted to the coronary care unit. *Archives of internal medicine*, *160*(12), 1875-1876.

Ikedo, F., Gangahar, D. M., Quader, M. A., & Smith, L. M. (2007). The effects of prayer, relaxation technique during general anesthesia on recovery outcomes following cardiac surgery. *Complementary Therapies in Clinical Practice*, *13*(2), 85-94.

Johnson, M. E., Dose, A. M., Pipe, T. B., Petersen, W. O., Huschka, M., Gallenberg, M. M., ... & Frost, M. H. (2009, July). Centering prayer for women receiving chemotherapy for recurrent ovarian cancer: a pilot study. In *Oncology nursing forum* (Vol. 36, No. 4).

Jonas, W. B. (2001). The middle way: realistic randomized controlled trials for the evaluation of spiritual healing.

Jones, B. M. (2001). Changes in cytokine production in healthy subjects practicing Guolin Qigong: a pilot study. *BMC complementary and alternative medicine*, *1*(1), 8.

Jorna, M., Ball, K., & Salmon, J. (2006). Effects of a holistic health program on women's physical activity and mental and spiritual health. *Journal of science and medicine in sport*, *9*(5), 395-401.

Kinjerski, V., & Skrypnek, B. J. (2008). The Promise of Spirit at WorkIncreasing Job Satisfaction and Organizational Commitment and Reducing Turnover and Absenteeism in Long-Term Care. *Journal of gerontological nursing*, *34*(10), 17-25.

Kozhevnikov, M., Louchakova, O., Josipovic, Z., & Motes, M. A. (2009). The enhancement of visuospatial processing efficiency through Buddhist deity meditation. *Psychological Science*, *20*(5), 645-653.

Krucoff, M. W., Crater, S. W., Gallup, D., Blankenship, J. C., Cuffe, M., Guarneri, M., ... & Pichard, A. (2005). Music, imagery, touch, and prayer as adjuncts to interventional cardiac care: the Monitoring and Actualisation of Noetic Trainings (MANTRA) II randomised study. *The Lancet*, *366*(9481), 211-217.

Lancaster, K. J., Schoenthaler, A. M., Midberry, S. A., Watts, S. O., Nulty, M. R., Cole, H. V., ... & Ogedegbe, G. (2014). Rationale and design of Faith-based Approaches in the Treatment of Hypertension (FAITH), a lifestyle intervention targeting blood pressure control among black church members. *American heart journal*, *167*(3), 301-307.

Leibovici, L. (2001). Effects of remote, retroactive intercessory prayer on outcomes in patients with bloodstream infection: randomised controlled trial. *Bmj*, *323*(7327), 1450-1451.

MacIntyre, B., Hamilton, J., Fricke, T., Ma, W., Mehle, S., & Michel, M. (2008). The efficacy of healing touch in coronary artery bypass surgery recovery: a randomized clinical trial. *Alternative therapies in health and medicine*, *14*(4), 24.

MacKinlay, E., & Trevitt, C. (2010). Living in aged care: Using spiritual reminiscence to enhance meaning in life for those with dementia. *International journal of mental health nursing*, *19*(6), 394-401.

Margolin, A., Avants, S. K., & Arnold, R. (2005). Acupuncture and spirituality-focused group therapy for the treatment of HIV-positive drug users: a preliminary study. *Journal of Psychoactive Drugs*, *37*(4), 385-390.

Mathai, J., & Bourne, A. (2004). Pilot study investigating the effect of intercessory prayer in the treatment of child psychiatric disorders. *Australasian Psychiatry*, *12*(4), 386-388.

Matthews, D. A., Marlowe, S. M., & MacNutt, F. S. (2000). Effects of intercessory prayer on patients with rheumatoid arthritis. *Southern medical journal*, *93*(12), 1177-1186.

Matthews, W. J., Conti, J. M., & Sireci, S. G. (2001). The effects of intercessory prayer, positive visualization, and expectancy on the well-being of kidney dialysis patients. *Alternative therapies in health and medicine*, *7*(5), 42.

McNabb, W., Quinn, M., Kerver, J., Cook, S., & Karrison, T. (1997). The PATHWAYS church-based weight loss program for urban African-American women at risk for diabetes. *Diabetes Care*, *20*(10), 1518-1523.

Moadel, A. B., Shah, C., Wylie-Rosett, J., Harris, M. S., Patel, S. R., Hall, C. B., & Sparano, J. A. (2007). Randomized controlled trial of yoga among a multiethnic sample of breast cancer patients: effects on quality of life. *Journal of Clinical Oncology*, *25*(28), 4387-4395.

Mok, E., Lau, K. P., Lai, T., & Ching, S. (2012, November). The meaning of life intervention for patients with advanced-stage cancer: development and pilot study. In *Oncology nursing forum* (Vol. 39, No. 6).

Nash, K. A. (2007). Implementation and evaluation of the empower youth program. *Journal of Holistic Nursing*, *25*(1), 26-36.

Newlin, K., Melkus, G. D., Peyrot, M., Koenig, H. G., Allard, E., & Chyun, D. (2010). Coping as a mediator in the relationships of spiritual well-being to mental health in black women with type 2 diabetes. *The International Journal of Psychiatry in Medicine*, *40*(4), 439-459.

Nidich, S. I., Fields, J. Z., Rainforth, M. V., Pomerantz, R., Cella, D., Kristeller, J., ... & Schneider, R. H. (2009). A randomized controlled trial of the effects of transcendental meditation on quality of life in older breast cancer patients. *Integrative Cancer Therapies*, *8*(3), 228-234.

Rummans, T. A., Clark, M. M., Sloan, J. A., Frost, M. H., Bostwick, J. M., Atherton, P. J., ... & Martensen, J. (2006). Impacting quality of life for patients with advanced cancer with a structured multidisciplinary intervention: a randomized controlled trial. *Journal of Clinical Oncology*, *24*(4), 635-642.

Samuel-Hodge, C. D., Keyserling, T. C., Park, S., Johnston, L. F., Gizlice, Z., & Bangdiwala, S. I. (2009). A randomized trial of a church-based diabetes self-management program for African Americans with type 2 diabetes. *The Diabetes Educator*, *35*(3), 439-454.

Scheel, I. B., Hagen, K. B., Herrin, J., Carling, C., & Oxman, A. D. (2002). Blind faith? The effects of promoting active sick leave for back pain patients: a cluster-randomized controlled trial. *Spine*, *27*(23), 2734-2740.

Shek, D. T. (2010). Objective outcome evaluation of the Project PATHS in Hong Kong: findings based on individual growth curve models. *The Scientific World Journal*, *10*, 182-191.

Sicher, F., Targ, E., Moore 2nd, D., & Smith, H. S. (1998). A randomized double-blind study of the effect of distant healing in a population with advanced AIDS. Report of a small scale study. *Western Journal of Medicine*, *169*(6), 356.

Steinhauser, K. E., Alexander, S. C., Byock, I. R., George, L. K., Olsen, M. K., & Tulsky, J. A. (2008). Do preparation and life completion discussions improve functioning and quality of life in seriously ill patients? Pilot randomized control trial. *Journal of palliative medicine*, *11*(9), 1234-1240.

Tsubono, K., Thomlinson, P., & Shealy, C. N. (2009). The effects of distant healing performed by a spiritual healer on chronic pain: a randomized controlled trial. *Alternative therapies in health and medicine*, *15*(3), 30.

Van der Does, W. (2000). A randomized, controlled trial of prayer?. *Archives of internal medicine*, *160*(12), 1871-1872.

VanLoon, P. (2009). The practice of interpersonal forgiveness in the personal and professional lives of clergy. *Journal of Pastoral Care & Counseling*, *63*(3-4), 1-9.

Walach, H., Bösch, H., Lewith, G., Naumann, J., Schwarzer, B., Falk, S., ... & Tomasson, H. (2008). Effectiveness of distant healing for patients with chronic fatigue syndrome: a randomised controlled partially blinded trial (EUHEALS). *Psychotherapy and psychosomatics*, *77*(3), 158-166.

Walker, S. R., Tonigan, J. S., Miller, W. R., Corner, S., & Kahlich, L. (1997). Intercessory prayer in the treatment of alcohol abuse and dependence: a pilot investigation. *Alternative Therapies in Health and Medicine*, *3*(6), 79-86.

Werch, C. E. C., Moore, M. J., Bian, H., DiClemente, C. C., Huang, I. C., Ames, S. C., ... & Pokorny, S. B. (2010). Are effects from a brief multiple behavior intervention for college students sustained over time?. *Preventive medicine*, *50*(1), 30-34.

Wiesendanger, H., Werthmüller, L., Reuter, K., & Walach, H. (2001). Chronically ill patients treated by spiritual healing improve in quality of life: results of a randomized waiting-list controlled study. *The Journal of Alternative & Complementary Medicine*, *7*(1), 45-51.

**Other methodology than randomized clinical trial: 39**

Ahmed, A. M. (2009). Are religious people more prosocial? A quasi‐experimental study with madrasah pupils in a rural community in India. *Journal for the Scientific Study of Religion*, *48*(2), 368-374.

Ando, M., Morita, T., Akechi, T., Ito, S., Tanaka, M., Ifuku, Y., & Nakayama, T. (2009). The efficacy of mindfulness-based meditation therapy on anxiety, depression, and spirituality in Japanese patients with cancer. *Journal of palliative medicine*, *12*(12), 1091-1094.

Balboni, T. A., Paulk, M. E., Balboni, M. J., Phelps, A. C., Loggers, E. T., Wright, A. A., ... & Prigerson, H. G. (2009). Provision of spiritual care to patients with advanced cancer: associations with medical care and quality of life near death. *Journal of Clinical Oncology*, *28*(3), 445-452.

Barlow, F., Lewith, G. T., & Walker, J. (2008). Experience of proximate spiritual healing in women with breast cancer, who are receiving long-term hormonal therapy. *The Journal of Alternative and Complementary Medicine*, *14*(3), 227-231.

Berger, R., & Gelkopf, M. (2009). School-based intervention for the treatment of tsunami-related distress in children: a quasi-randomized controlled trial. *Psychotherapy and psychosomatics*, *78*(6), 364-371.

Bloom, J. R., Stewart, S. L., D’Onofrio, C. N., Luce, J., & Banks, P. J. (2008). Addressing the needs of young breast cancer survivors at the 5 year milestone: can a short-term, low intensity intervention produce change?. *Journal of Cancer Survivorship*, *2*(3), 190-204.

Brown, A. E., Pavlik, V. N., Shegog, R., Whitney, S. N., Friedman, L. C., Romero, C., ... & Volk, R. J. (2007). Association of spirituality and sobriety during a behavioral spirituality intervention for twelve step (TS) recovery. *The American journal of drug and alcohol abuse*, *33*(4), 611-617.

Brown, C. G., Mory, S. C., Williams, R., & McClymond, M. J. (2010). Study of the therapeutic effects of proximal intercessory prayer (STEPP) on auditory and visual impairments in rural Mozambique. *Southern medical journal*, *103*(9), 864-869.

Byrd, R. C. (1988). Positive therapeutic effects of intercessory prayer in a coronary care unit population. *Southern medical journal*, *81*(7), 826-829.

Carlson, L. E., Speca, M., Faris, P., & Patel, K. D. (2007). One year pre–post intervention follow-up of psychological, immune, endocrine and blood pressure outcomes of mindfulness-based stress reduction (MBSR) in breast and prostate cancer outpatients. *Brain, behavior, and immunity*, *21*(8), 1038-1049.

Chi, F. W., Kaskutas, L. A., Sterling, S., Campbell, C. I., & Weisner, C. (2009). Twelve‐step affiliation and 3‐year substance use outcomes among adolescents: Social support and religious service attendance as potential mediators. *Addiction*, *104*(6), 927-939.

Cole, B., Broer, K., Hopkins, C., Tisak, J., Hunt, R., McNally, C., ... & Boyiadzis, M. (2010). A Randomized Controlled Trial of Spiritually-Focused Meditation In Patients Newly Diagnosed with Acute Leukemia. *Blood*, *116*(21), 1519-1519.

Cunningham, A. J., & Tocco, E. K. (1989). A randomized trial of group psychoeducational therapy for cancer patients. *Patient Education and Counseling*, *14*(2), 101-114.

Daly, B. J., Douglas, S. L., Gunzler, D., & Lipson, A. R. (2013). Clinical trial of a supportive care team for patients with advanced cancer. *Journal of pain and symptom management*, *46*(6), 775-784.

Deckro, G. R., Ballinger, K. M., Hoyt, M., Wilcher, M., Dusek, J., Myers, P., ... & Benson, H. (2002). The evaluation of a mind/body intervention to reduce psychological distress and perceived stress in college students. *Journal of American College Health*, *50*(6), 281-287.

Delaney, C., & Barrere, C. (2009). Ecospirituality: The experience of environmental meditation in patients with cardiovascular disease. *Holistic nursing practice*, *23*(6), 361-369.

Delaney, H. D., Forcehimes, A. A., Campbell, W. P., & Smith, B. W. (2009). Integrating spirituality into alcohol treatment. *Journal of Clinical Psychology*, *65*(2), 185-198.

Erwin, D. O., Spatz, T. S., Stotts, R. C., & Hollenberg, J. A. (1999). Increasing mammography practice by African American women. *Cancer practice*, *7*(2), 78-85.

Eshah, N. F., Bond, A. E., & Froelicher, E. S. (2010). The effects of a cardiovascular disease prevention program on knowledge and adoption of a heart healthy lifestyle in Jordanian working adults. *European Journal of Cardiovascular Nursing*, *9*(4), 244-253.

Fisch, M. J., Titzer, M. L., Kristeller, J. L., Shen, J., Loehrer, P. J., Jung, S. H., ... & Einhorn, L. H. (2003). Assessment of quality of life in outpatients with advanced cancer: the accuracy of clinician estimations and the relevance of spiritual well-being—a Hoosier Oncology Group study. *Journal of Clinical Oncology*, *21*(14), 2754-2759.

Ford, M. E., Edwards, G., Rodriguez, J. L., Gibson, R. C., & Tilley, B. C. (1996). An empowerment-centered, church-based asthma education program for African American adults. *Health and Social Work*, *21*(1), 70.

Garland, S. N., Carlson, L. E., Cook, S., Lansdell, L., & Speca, M. (2007). A non-randomized comparison of mindfulness-based stress reduction and healing arts programs for facilitating post-traumatic growth and spirituality in cancer outpatients. *Supportive Care in Cancer*, *15*(8), 949-961.

Glueckauf, R. L., Davis, W. S., Allen, K., Chipi, P., Schettini, G., Tegen, L., ... & Prescott, S. (2009). Integrative cognitive–behavioral and spiritual counseling for rural dementia caregivers with depression. *Rehabilitation psychology*, *54*(4), 449.

Helm, H. M., Hays, J. C., Flint, E. P., Koenig, H. G., & Blazer, D. G. (2000). Does private religious activity prolong survival? A six-year follow-up study of 3,851 older adults. *The Journals of Gerontology Series A: Biological Sciences and Medical Sciences*, *55*(7), M400-M405.

Kim, K. H. C., Linnan, L., Kramish Campbell, M., Brooks, C., Koenig, H. G., & Wiesen, C. (2008). The WORD (wholeness, oneness, righteousness, deliverance): a faith-based weight-loss program utilizing a community-based participatory research approach. *Health Education & Behavior*, *35*(5), 634-650.

Knight, J. R., Sherritt, L., Harris, S. K., Holder, D. W., Kulig, J., Shrier, L. A., ... & Chang, G. (2007). Alcohol use and religiousness/spirituality among adolescents. *Southern Medical Journal*, *100*(4), 349.

Kretzer, K., Davis, J., Easa, D., Johnson, J., & Harrigan, R. (2007). Self identity through Ho'oponopono as adjunctive therapy for hypertension management. *Ethnicity and Disease*, *17*(4), 624.

Kumar, S. P., Jim, A., & Sisodia, V. (2011). Effects of palliative care training program on knowledge, attitudes, beliefs and experiences among student physiotherapists: A preliminary quasi-experimental study. *Indian journal of palliative care*, *17*(1), 47.

Moskowitz, J. T., Hult, J. R., Duncan, L. G., Cohn, M. A., Maurer, S., Bussolari, C., & Acree, M. (2012). A positive affect intervention for people experiencing health-related stress: Development and non-randomized pilot test. *Journal of Health Psychology*, *17*(5), 676-692.

Phelps, A. C., Maciejewski, P. K., Nilsson, M., Balboni, T. A., Wright, A. A., Paulk, M. E., ... & Prigerson, H. G. (2009). Religious coping and use of intensive life-prolonging care near death in patients with advanced cancer. *Jama*, *301*(11), 1140-1147.

Rentala, S., Fong, T. C., Nattala, P., Chan, C. L., & Konduru, R. (2015). Effectiveness of body–mind–spirit intervention on well‐being, functional impairment and quality of life among depressive patients–a randomized controlled trial. *Journal of advanced nursing*, *71*(9), 2153-2163.

Rungreangkulkij, S., & Wongtakee, W. (2008). The psychological impact of Buddhist counseling for patients suffering from symptoms of anxiety. *Archives of psychiatric nursing*, *22*(3), 127-134.

Rushton, C. H., Sellers, D. E., Heller, K. S., Spring, B., Dossey, B. M., & Halifax, J. (2009). Impact of a contemplative end-of-life training program: Being with dying. *Palliative and Supportive Care*, *7*(04), 405-414.

Scherwitz, L., Pullman, M., McHenry, P., Gao, B., & Ostaseski, F. (2006). A contemplative care approach to training and supporting hospice volunteers: A prospective study of spiritual practice, well-being, and fear of death. *Explore: The Journal of Science and Healing*, *2*(4), 304-313.

Tarakeshwar, N., Pearce, M. J., & Sikkema, K. J. (2005). Development and implementation of a spiritual coping group intervention for adults living with HIV/AIDS: A pilot study. *Mental Health, Religion & Culture*, *8*(3), 179-190.

Vuckovic, N., Schneider, J., Williams, L. A., & Ramirez, M. (2010). Journey into healing: the transformative experience of shamanic healing on women with temporomandibular joint disorders. *Explore: The Journal of Science and Healing*, *6*(6), 371-379.

Wall, R. J., Engelberg, R. A., Gries, C. J., Glavan, B., & Curtis, J. R. (2007). Spiritual care of families in the intensive care unit. *Critical care medicine*, *35*(4), 1084-1090.

Whitt-Glover, M. C., Hogan, P. E., Lang, W., & Heil, D. P. (2008). Pilot study of a faith-based physical activity program among sedentary blacks. *Prev Chronic Dis*, *5*(2), A51.

Zemore, S. E. (2007). A role for spiritual change in the benefits of 12‐step involvement. *Alcoholism: Clinical and Experimental Research*, *31*(s3), 76s-79s.

**Not adequate randomization: 16**

Andrews JO, Felton G, Ellen Wewers M, Waller J, Tingen M. The effect of a multi‐component smoking cessation intervention in African American women residing in public housing. *Research in nursing & health*. 2007;30(1):45-60.

Harris JI, Erbes CR, Engdahl BE, Thuras P, Murray‐Swank N, Grace D, Malec C. The effectiveness of a trauma focused spiritually integrated intervention for veterans exposed to trauma. *Journal of clinical psychology*. 2011;67(4):425-38.

Holt CL, Klem PR. As you go, spread the word: spiritually based breast cancer education for African American women. *Gynecologic oncology*. 2005;99(3):S141-S142.

Ka'opua LSI, Park SH, Ward ME, Braun KL. Testing the feasibility of a culturally tailored breast cancer screening intervention with Native Hawaiian women in rural churches. *Health Soc Work*. 2011;36(1):55-65.

Kim KHC, Linnan L, Kramish Campbell M, Brooks C, Koenig HG, Wiesen C. The WORD (wholeness, oneness, righteousness, deliverance): a faith-based weight-loss program utilizing a community-based participatory research approach. *Health Education & Behavior*. 2008;35(5):634-50.

Liu CJ, Hsiung PC, Chang KJ, Liu YF, Wang KC, Hsiao FH, Chan CL. A study on the efficacy of body–mind–spirit group therapy for patients with breast cancer. *Journal of clinical nursing*. 2008;17(19):2539-49.

Miller JJ, Frost MH, Rummans TA, Huschka M, Atherton P, Brown P, Clark MM. Role of a medical social worker in improving quality of life for patients with advanced cancer with a structured multidisciplinary intervention. *Journal of psychosocial oncology*. 2007;25(4):105-19.

Moeini M, Ghasemi TMG, Yousefi H, Abedi H. The effect of spiritual care on spiritual health of patients with cardiac ischemia. *Iranian journal of nursing and midwifery research*. 2012;17(3):195.

Mosalanejad L, Koolee AK. Looking at infertility treatment through the lens of the meaning of life: the effect of group logotherapy on psychological distress in infertile women. *Int J Fertil Steril*. 2013;6(4).

Petry NM, Lewis MW, Østvik-White EM. Participation in religious activities during contingency management interventions is associated with substance use treatment outcomes. *American Journal on Addictions*. 2008;17(5):408-13.

Rosendahl J, Tigges-Limmer K, Gummert J, Dziewas R, Albes JM, Strauss B. Bypass surgery with psychological and spiritual support (the By. pass study): study design and research methods. *Am Heart J*. 2009;158(1):8-14.

Targ EF, Levine EG. The efficacy of a mind-body-spirit group for women with breast cancer: a randomized controlled trial. *General Hospital Psychiatry*. 2002;24(4):238-48.

Warber SL, Ingerman S, Moura VL, Wunder J, Northrop A, Gillespie BW, Rubenfire M. Healing the heart: a randomized pilot study of a spiritual retreat for depression in acute coronary syndrome patients. *EXPLORE: The Journal of Science and Healing*. 2011;7(4):222-33.

Williams LB, Sattin RW, Dias J, Garvin JT, Marion L, Joshua T, Narayan KV. Design of a cluster-randomized controlled trial of a diabetes prevention program within African–American churches: the fit body and soul study. *Contemporary clinical trials*. 2013;34(2): 336-47.

Yoshimoto SM, Ghorbani S, Baer JM, Cheng KW, Banthia R, Malcarne VL, Varni JW. Religious coping and problem‐solving by couples faced with prostate cancer. *European Journal of Cancer Care*. 2006;15(5):481-8.

Zhang H, Neelarambam K, Schwenke TJ, Rhodes MN, Pittman DM, Kaslow NJ. Mediators of a culturally-sensitive intervention for suicidal African American women. *Journal of clinical psychology in medical settings*. 2013;20(4):401-14.

**Mental Health: 15**

# Babamohamadi H, Sotodehasl N, Koenig HG, Jahani C, Ghorbani R. The effect of Holy Qur’an recitation on anxiety in hemodialysis patients: A randomized clinical trial. *J Relig and Health*. 2015;54(5):1921-30.

Bay PS, Beckman D, J Trippi, Gunderman R, Terry C. The effect of pastoral care services on anxiety, depression, hope, religious coping, and religious problem solving styles: A randomized controlled study. *J Relig and Health.* 2008;47:57-69.

Bowland S, Edmond T, Fallot RD. Evaluation of a spiritually focused intervention with older trauma survivors. *Social Work*. 2012;57(1):73-82.

Breitbart W, RosenfelD B, Gibson C, Pessin H, Poppito S, Nelson C, Olden M. Meaning centered group psychotherapy for patients with advanced cancer: a pilot randomized controlled trial. *Psycho Oncology.* 2010;19(1):21-8.

# Ebrahimi H, Kazemi AH, Khoshknab MF, Modabber R. The effect of spiritual and religious group psychotherapy on suicidal ideation in depressed patients: a randomized clinical trial. *J of Caring Scie*. 2014;3(2):131.

# Hosseini M, Salehi A, Fallahi Khoshknab M, Rokofian A, Davidson PM. The effect of a preoperative spiritual/religious intervention on anxiety in Shia Muslim patients undergoing coronary artery bypass graft surgery: a randomized controlled trial. *J Holist Nurs.* 2013;31(3):134-72.

Huguelet P, Mohr S, Betrisey C, Borras L, Gillieron C, Marie AM, Rieben I, Perroud N, Brandt PY. A randomized trial of spiritual assessment of outpatients with schizophrenia: patients' and clinicians' experience. *Psychiatr Serv.* 2011;62(1): 79-86.

Kelly JF, Stout RL, Magilli M, Tonigan JS, Pagano ME. Spirituality in recovery: a lagged mediational analysis of alcoholics anonymous' principal theoretical mechanism of behavior change. *Alcohol Clin Exp Res.* 2011;35(3):454-63.

Koszycki D, Bilodeau C, Raab-Mayo K, Bradwejn J. A multifaith spiritually based intervention versus supportive therapy for generalized anxiety disorder: a pilot randomized trial. Epub 2013. Disponível em: http://onlinelibrary.wiley.com/doi/10.1002/jclp.22052/pdf. Acesso em 20 out. 2013.

Llyod-Williams M, Cobb M, O´Connor C, Dunn L, Shiels C. A pilot randomized controlled trial to reduce suffering and emotional distress in patients with advanced cancer. *Journal Affective Disorders*. 2013;148(1):141-5.

Miller DK, Chibnall JT, Videen SD, Duckro PN. Supportive-affective group experience for persons with life-threatening illness: reducing spiritual, psychological, and death-related distress in dying patients. *J Palliat Med*. 2005;8(2):333-43.

Miller WR, Forcehimes A,O'Leary MJ, LaNoue MD. Spiritual direction in addiction treatment: two clinical trials. *J Subst Abuse Treat*. 2008;35(4):434-42.

Moeini M, Taleghani F, Mehrabi T, Musarezaie A. Effect of a spiritual care program on levels of anxiety in patients with leukemia. *Iranian journal of nursing and midwifery research*. 2014;19(1):88.

Moritz S, Quan H, Rickhi B, Liu M, Angen M, Vintila R, Sawa R, Soriano J, Toews J. A home study-based spirituality education program decreases emotional distress and increases quality of life-a randomized, controlled trial. *Altern Ther Health Med.* 2006;12(6):26-35.

Rickhi B, Moritz S, Reesal R, Xu TJ, Paccagnan P, Urbanska B, Quan H. A Spirituality Teaching Program for Depression: A Randomized Controlled Trial. *Int J Psychiatry Med*. 2011;42(3):315-29.

**Without return of email: 15**

Azhar MZ, Varma SL, Dharap AS. Religious psychotherapy in anxiety disorder patients. *Acta Psychiatrica Scandinavica*.1994;90(1):1-3.

Azhar MZ, Varma SL. Religious psychotherapy in depressive patients. *Psychotherapy and psychosomatics*.1995;63(3-4):165-8.

Bay PS, Ivy SS, Terry CL. The effect of spiritual retreat on nurses' spirituality: a randomized controlled study. *Holistic nursing practice*. 2010;24(3):125-33.

Chan CH, Chan CL, Ng EH, Ho PC, Chan TH, Lee GL, Hui WHC. Incorporating spirituality in psychosocial group intervention for women undergoing in vitro fertilization: a prospective randomized controlled study. *Psychology and Psychotherapy: Theory, Research and Practice*. 2012;85(4):356-73.

Chen YY. Written emotional expression and religion: effects on PTSD symptoms. *The International Journal of Psychiatry in Medicine*. 2005;35(3):273-86.

Holt CL, Wynn TA, Southward P, Litaker MS, Jeames S, Schulz E. Development of a spiritually based educational intervention to increase informed decision making for prostate cancer screening among church-attending African American men. *Journal of health communication*. 2009;14(6):590-604.

Koszycki D, Raab K, Aldosary F, Bradwejn J. A multifaith spiritually based intervention for generalized anxiety disorder: a pilot randomized trial. *J Clin Psychol.* 2010;66(4):430-41.

# Morgan PD, Fogel J, Tyler ID, Jones JR. Culturally targeted educational intervention to increase colorectal health awareness among African Americans. *Journal of health care for the poor and underserved*. 2010;21(3):132-47.

# Piderman KM, Johnson ME. Hospital chaplains' involvement in a randomized controlled multidisciplinary trial: implications for spiritual care and research. *Journal of Pastoral Care & Counseling*. 2009;63(3-4):1-6.

# Razali SM, Hasanah CI, Aminah K, Subramaniam M. Religious—sociocultural psychotherapy in patients with anxiety and depression. Australian and New Zealand *Journal of Psychiatry*. 1998;32(6):867-72.

# Razali SM, Aminah K, Khan UA. Religious–cultural psychotherapy in the management of anxiety patients. *Transcultural Psychiatry*. 2002;39(1):130-6.

# Rye MS, Pargament KI, Pan W, Yingling DW, Shogren KA, Ito M. Can group interventions facilitate forgiveness of an ex-spouse? A randomized clinical trial. *Journal of consulting and clinical psychology*. 2005;73(5):880.

# Scott Richards P, Berrett ME, Hardman RK, Eggett DL. Comparative efficacy of spirituality, cognitive, and emotional support groups for treating eating disorder inpatients. *Eating Disorders*. 2006;14(5):401-15.

# Stahler GJ, Kirby KC, Kerwin ME. A faith-based intervention for cocaine-dependent Black women. *Journal of psychoactive drugs*. 2007;39(2):183-90.

# Voorhees CC, Stillman FA, Swank RT, Heagerty PJ, Levine DM, Becker DM. Heart, body, and soul: impact of church-based smoking cessation interventions on readiness to quit. *Preventive medicine*. 1996;25(3):277-85.

**Inadequate: 1**

Holt CL, Wynn TA, Litaker MS, Southward P, Jeames SE, Schulz EK. A comparison of a spiritually based and non-spiritually based educational intervention for informed decision making for prostate cancer screening among church-attending African-American men. *Urologic nursing*. 2009;29(4):249.
