# Supplementary material for: Complementary religious and spiritual interventions in physical health and quality of life: A systematic review of randomized controlled clinical trials
Source: PLoS One. 2017 Oct 19;12(10):e0186539. doi: 10.1371/journal.pone.0186539 (PMC5648186; doi:10.1371/journal.pone.0186539)
Supplement: S3 File — Boolean expression with the key words used for the search of the articles are presented for each database used. (DOCX) [file pone.0186539.s003.docx]

**Search Strategy for PubMed**

Boolean expression used in main search:

(spiritu* OR relig* OR Faith OR holistic OR multifaith) AND (assistance OR intervention OR treatment OR therapy OR assessment OR group OR meditation) AND (clinical trial OR meta-analysis OR Randomized controlled trial OR controlled clinical trial)

No filters used.

**Search Strategy for Web of Science**

Boolean expression used in main search:

(spiritu* OR relig* OR Faith OR holistic OR multifaith) AND (assistance OR intervention OR treatment OR therapy OR assessment OR group OR meditation) AND (clinical trial OR meta-analysis OR Randomized controlled trial OR controlled clinical trial)

No filters used.

**Search Strategy for Scopus**

Boolean expression used in main search:

(spiritu* OR relig* OR Faith OR holistic OR multifaith) AND (assistance OR intervention OR treatment OR therapy OR assessment OR group OR meditation) AND (clinical trial OR meta-analysis OR Randomized controlled trial OR controlled clinical trial)

No filters used.

**Search Strategy for Embase**

Boolean expression used in main search:

(spiritu* OR relig* OR Faith OR holistic OR multifaith) AND (assistance OR intervention OR treatment OR therapy OR assessment OR group OR meditation) AND (clinical trial OR meta-analysis OR Randomized controlled trial OR controlled clinical trial)

Filters used:

FAZER LOGIN PARA REVER OS FILTROS…

**Search Strategy for PsychINFO**

Boolean expression used in main search:

(spiritu* OR relig* OR Faith OR holistic OR multifaith) AND (assistance OR intervention OR treatment OR therapy OR assessment OR group OR meditation) AND (clinical trial OR meta-analysis OR Randomized controlled trial OR controlled clinical trial)

No filters used.

**Search Strategy for The Cochrane Collaboration Library**

Boolean expression used in main search:

(spiritu* OR relig* OR Faith OR holistic OR multifaith) AND (assistance OR intervention OR treatment OR therapy OR assessment OR group OR meditation) AND (clinical trial OR meta-analysis OR Randomized controlled trial OR controlled clinical trial)

Filters used: Trials.

**Search Strategy for Scielo**

Boolean expression used in main search:

(spiritu* OR relig* OR Faith OR holistic OR multifaith) AND (assistance OR intervention OR treatment OR therapy OR assessment OR group OR meditation) AND (clinical trial OR meta-analysis OR Randomized controlled trial OR controlled clinical trial)

No filters used.
