# Supplementary material for: Complementary religious and spiritual interventions in physical health and quality of life: A systematic review of randomized controlled clinical trials
Source: PLoS One. 2017 Oct 19;12(10):e0186539. doi: 10.1371/journal.pone.0186539 (PMC5648186; doi:10.1371/journal.pone.0186539)
Supplement: S1 Table — (DOCX) [file pone.0186539.s004.docx]

**S1 Table. Characteristics of religious and spiritual interventions in others outcomes.**

| **Author** | **Population/**  **Condition** | **Sample Size** | **Type of Intervention** | **Focus of Intervention** | **Facilitators** | **Sessions/**  **Duration (min)** | **Control Groups** | **Follow Up (months)** | **Outcomes and Results Assessed  (Cohen d [IC:95%])** | **Score** |
| --- | --- | --- | --- | --- | --- | --- | --- | --- | --- | --- |
| Bormann, 2009 | HIV+ | 93 | Meditation | Spiritual | Authors | 5/ 90 | EdCG | 1 to 6 | Cortisol Post-treatment: d=0.28 [-0.75 ; 0.19] Cortisol 1-month: d=0.04 [-0.43 ; 0.50] | 8 |
| Duru, 2010 | Sedentary | 62 | Church | Religious | Research assistant | 8/ 90 | EdCG | 1 to 6 | SBP: *MD=-11.0, (SD=3.9) (p=0.007) DBP: *MD=-2.0, (SD=3.0) (p=0.48) | 8 |
| Guilherme, 2016 | Breast cancer with mastectomy | 27 | Guided visualization | Spiritual | Authors | 1/ N/M | TCG | < 1 | HR: d=0.91 [-1.70 ; -0.11] | 7 |
| Koenig, 2015 | Chronic medical illness and depression | 132 | Psychotherapy | Religious | Psychologists post-graduate | 10/ 50 | TCG | 1 to 6 | DASI: d=0.09 [-0.43 ; 0.26] | 9 |
| McCauley, 2011 | Chronic pain | 100 | Audiovisual | Spiritual | Material made by authors | 5/ 28 | EdCG | 1 to 6 | Energy (MOS): d=0.18 [-0.24 ; 0.61] SEMCD: d=0.19 [-0.24 ; 0.62] | 9 |

Legend: EdCG=Educational Control Group; TCG=Therapeutic Control Group; SBP=systolic blood pressure; DBP=diastolic blood pressure; HR=Heart Rate; DASI=Daily Activity Status Index; MOS=Medical Outcome Study in the Chronic Disease Self-Management study; SEMCD=Self-Efficacy to Manage Chronic Diseases.
*MD=Mean Difference (SD=Standard Deviation), article without data to calculate Cohen d.
