# Supplementary material for: Complementary religious and spiritual interventions in physical health and quality of life: A systematic review of randomized controlled clinical trials
Source: PLoS One. 2017 Oct 19;12(10):e0186539. doi: 10.1371/journal.pone.0186539 (PMC5648186; doi:10.1371/journal.pone.0186539)
Supplement: S2 Table — (DOCX) [file pone.0186539.s005.docx]

**S2 Table. Characteristics of religious and spiritual interventions in the promotion of healthy behavior outcomes.**

| **Author** | **Population/Condition** | **Sample Size** | **Type of Intervention** | **Focus of Intervention** | **Facilitators** | **Sessions/Duration (min)** | **Control Groups** | **Follow Up (months)** | **Outcomes and Results Assessed  (Cohen d [IC:95%])** | **Score** |
| --- | --- | --- | --- | --- | --- | --- | --- | --- | --- | --- |
| Arriola, 2010 | Healthy | 425 | Church | Religious | Material made by authors | > 1/ N/M | EdCG | > 6 | Donation related-video: d=0.63 [0.42 ; 0.82] Donation written material: d=0.33 [0.14 ; 0.53] | 6 |
| Holt, 2008 | Healthy | 108 | Church | Religious | Authors | 1/ N/M | EdCG | < 1 | Personal connection: d=0.94 [0.53 ; 1.35] Self-assessment: d=0.85 [0.45 ; 1.25] Behavior intention: d=0.10 [-0.26 ; 0.48] Ideas about health impacts: d=0.22 [-0.15 ; 0.04] | 8 |
| Holt, 2012 | Healthy | 316 | Church | Religious | Church leaders and authors | 2/ N/M | EdCG | < 1 | CRSB: d=0.22 [-0.05 ; 0.48] FOBT PBe: d=0.04 [-0.22 ; 0.30] FOBT PBa: d=0.09 [-0.17 ; 0.36] C PBe: d=0.19 [-0.07 ; 0.46] C PBa: d=0.01 [-0.28 ; 0.25] | 6 |
| Margolin, 2006 | Drug users | 72 | Psychotherapy | Spiritual | Authors | 8/ 60 | TCG | 1 to 6 | MP HIV: d=1.02 [0.49 ; 1.56] HIV RB: d=0.65 [0.12 ; 1.18] | 6 |
| Wingood, 2013 | HIV+ | 118 | Psychotherapy | Religious | Church members | 3/ 45 | EdCG | 1 to 6 | CCU: *MD=-0.49 [-1.31 ; 0.33] CULS: *MD=-0.17 [-0.75 ; 0.41] NCU: *MD=-0.11 [-0.71 ; 0.49] SA: *MD=-0.13 [-0.61 ; 0.36] | 5 |

Legend: EdCG=Educational Control Group; TCG=Therapeutic Control Group; CRSB=Colorectal Cancer Screening Benefits; FOBT PBe=Fecal Occult Blood Test Perceived Benefits; FOBT PBa=Fecal Occult Blood Test Perceived Barriers; C PBe=Colonoscopy Perceived Benefits; C PBa=Colonoscopy Perceived Barriers; MP HIV=Motivation to Prevent HIV; HIV RB=HIV Risk Behavior from The Risk Assessment Battery; CCU=Consistent Condom Use (past 90 days); CULS=Condom Use at Last Sex; NCU=Never Condom Use (past 30 days); SA=Sex Abstinence (>30 days).
*MD=Mean Difference, article without data to calculate Cohen d.
