# Supplementary material for: Complementary religious and spiritual interventions in physical health and quality of life: A systematic review of randomized controlled clinical trials
Source: PLoS One. 2017 Oct 19;12(10):e0186539. doi: 10.1371/journal.pone.0186539 (PMC5648186; doi:10.1371/journal.pone.0186539)
Supplement: S3 Table — (DOCX) [file pone.0186539.s006.docx]

**S3 Table. Characteristics of religious and spiritual interventions in clinical practice outcomes of health professionals.**

| **Author** | **Population/Condition** | **Sample Size** | **Type of Intervention** | **Focus of Intervention** | **Facilitators** | **Sessions/Duration (min)** | **Control Groups** | **Follow Up (months)** | **Outcomes and Results Assessed  (Cohen d [IC:95%])** | **Score** |
| --- | --- | --- | --- | --- | --- | --- | --- | --- | --- | --- |
| Burkhart, 2012 | Nurses | 59 | Psychotherapy | Spiritual | Authors | 2/ N/M | EdCG | < 1 | SCI: *MD=5.7 (p<0.01) SCIP: *MD=4.0 (p<0.05) | 5 |
| Morita, 2009 | Nurses | 41 | Psychotherapy | Spiritual | Authors | 8/ 180 | WLG | > 6 | Confidence: **DP=51% (p<0.001)  SRPS: **DP=12% (p<0.001) ACPFMS: **DP=5.6% (p=0.028) MBS: **DP=-21% (p<0.001) | 6 |
| Oman, 2006 | Nurses, physicians, chaplains, psychologists, physical and occupational therapist | 58 | Meditation | Spiritual | Authors | 5/ 90 | WLG | 1 to 6 | Job Satisfaction: d=0.08 [-0.44 ; 0.60] EE (MBS): d=1.0 [-4.33 ; 2.33] PA (MBS): d=1.51 [-0.38 ; 3.40] | 7 |
| Oman, 2008 | Nurses, physicians, chaplains, psychologists, physical and occupational therapist | 58 | Meditation | Spiritual | Authors | 5/ 90 | WLG | 1 to 6 | RCSES: d=0.40 [0.08 ; 0.72] | 7 |
| Legend: EdCG=Educational Control Group; WLG=Waiting List Group; SCI=Spiritual care Inventory; SCIP=Spiritual Care in Practice; SRPS=Self-Reported Practice Scale; ACPFMS=Attitudes toward Caring for Patients Feeling Meaningless Scale; MBS=Maslach Burnout Scale; EE=Emotional Exhaustion; PA=Personal Accomplishment; RCSES=Relational Caregiving Self-Efficacy Scale. *MD=Mean Difference, article without Standard Deviation to calculate Cohen d. **DP=Difference Proportion, article without data to calculate Cohen d. | | | | | | | | | | |

Legend: EdCG=Educational Control Group; WLG=Waiting List Group; SCI=Spiritual care Inventory; SCIP=Spiritual Care in Practice; SRPS=Self-Reported Practice Scale; ACPFMS=Attitudes toward Caring for Patients Feeling Meaningless Scale; MBS=Maslach Burnout Scale; EE=Emotional Exhaustion; PA=Personal Accomplishment; RCSES=Relational Caregiving Self-Efficacy Scale.
*MD=Mean Difference, article without Standard Deviation to calculate Cohen d.
**DP=Difference Proportion, article without data to calculate Cohen d.
